# Construction and optimization of non-parametric analysis model for meter coefficients via back propagation neural network

**DOI:** 10.1038/s41598-024-61702-2

**Published:** 2024-05-20

**Authors:** Yuqiang Yang, Ruoyun Hu, Weifeng Wang, Tuomu Zhang

**Affiliations:** 1 State Grid Zhejiang Electric Power Co. Ltd, Hanzghou City, 310007 China; 2 Department of Marketing, State Grid Zhejiang Electric Power Co. Ltd, Hanzghou City, 310007 China; 3 Beijing Zhixiang Technology Co., Ltd., Beijing City, 100000 China

**Keywords:** Meter coefficient analysis, BP neural network, Deep learning technology, Non-parametric analysis, Energy metering, Energy science and technology, Engineering

## Abstract

This study addresses the drawbacks of traditional methods used in meter coefficient analysis, which are low accuracy and long processing time. A new method based on non-parametric analysis using the Back Propagation (BP) neural network is proposed to overcome these limitations. The study explores the classification and pattern recognition capabilities of the BP neural network by analyzing its non-parametric model and optimization methods. For model construction, the study uses the United Kingdom Domestic Appliance-Level Electricity dataset’s meter readings and related data for training and testing the proposed model. The non-parametric analysis model is used for data pre-processing, feature extraction, and normalization to obtain the training and testing datasets. Experimental tests compare the proposed non-parametric analysis model based on the BP neural network with the traditional Least Squares Method (LSM). The results demonstrate that the proposed model significantly improves the accuracy indicators such as mean absolute error (MAE) and mean relative error (MRE) when compared with the LSM method. The proposed model achieves an MAE of 0.025 and an MRE of 1.32% in the testing dataset, while the LSM method has an MAE of 0.043 and an MRE of 2.56% in the same dataset. Therefore, the proposed non-parametric analysis model based on the BP neural network can achieve higher accuracy in meter coefficient analysis when compared with the traditional LSM method. This study provides a novel non-parametric analysis method with practical reference value for the electricity industry in energy metering and load forecasting.

## Introduction

Neural networks have been extensively used in various fields, such as pattern recognition, signal processing, prediction, and classification in recent years due to their non-linear mapping technology^1,2^. Among the neural network models, Back Propagation (BP) is the most commonly used model and is widely applied in industries such as electric power, water conservancy, transportation, finance, and others. The BP neural network is favored due to its high adaptability, short training time, and excellent prediction accuracy, providing an effective solution for practical engineering problems. However, it still has some issues, including limited recognition ability and the tendency to fall into local optima, which affect the real-time and accuracy requirements of industrial applications when compared to traditional methods. This study aims to address the aforementioned problems by improving the measurement accuracy and stability of electric meters. A non-parametric analysis model suitable for electric meter coefficients is proposed, and neural network optimization algorithms are employed to improve the BP neural network’s application effect in electric power measurement^3^. In addition, the non-parametric analysis model’s advantages and feasibility in electric power measurement are explored to develop a new method applicable to practical industrial problems.

Electric meters play a crucial role in the electric power system as they are used for measuring and monitoring energy consumption. The accuracy and stability of electric meters have a direct impact on the safety and operational efficiency of the system. However, traditional electric meter testing methods that rely on parameterized analytical models, such as three-phase three-wire and three-phase four-wire, have been found to have low accuracy and large model errors^4,5^. In contrast, non-parametric analysis models are known for their high reliability, strong adaptability, and high accuracy.

Accurate analysis of meter coefficients plays a pivotal role in effective energy management and power consumption monitoring within the electricity industry. Traditional methods, characterized by low accuracy and prolonged processing times, primarily rely on parametric models. These models entail assumptions about data distribution and involve intricate data preprocessing and feature extraction procedures, making them susceptible to noise and outliers, ultimately diminishing analysis accuracy. Furthermore, these methods demand substantial human and time resources, struggling to handle extensive data and intricate patterns. Recognizing the imperative need for precise meter coefficient analysis in the electricity industry, there has been a growing interest in non-parametric analysis methods and neural network technologies. Non-parametric methods, by avoiding assumptions about data distributions, leverage data characteristics directly, providing greater applicability and flexibility. Widely utilized in pattern recognition and classification, non-parametric methods excel in identifying intricate patterns and achieving precise classification. In contrast, neural networks, as potent pattern recognition tools, possess robust nonlinear fitting capabilities and excel in handling large-scale data. This has led to their widespread adoption as solutions to challenges faced by traditional methods. Neural networks offer significant advantages in constructing non-parametric analysis models. Firstly, they adeptly handle substantial datasets, autonomously learning complex patterns and features through deep learning, eliminating the need for manually designed feature extractors. Secondly, neural networks exhibit excellent adaptability, automatically adjusting model parameters in response to data changes, thereby accommodating various data analysis tasks in terms of types and scales. Additionally, the flexible nature of neural network structures allows for adjustments tailored to specific problems, thereby enhancing model performance and adaptability. Against this backdrop, this study proposes a novel non-parametric analysis model based on BP neural networks to overcome the limitations of traditional meter coefficient analysis methods. This study endeavors to capitalize on the strengths of neural networks to elevate the accuracy and efficiency of meter coefficient analysis, presenting more effective tools for energy management and power consumption monitoring in the electricity industry. Through this model, the objective is to achieve heightened accuracy, efficiency, and reliability in meter coefficient analysis, contributing significantly to the development and sustainability of the electricity industry. The study analyzes non-parametric models and electric energy metering technology and employs regularization or dropout techniques to optimize the model during the BP neural network model building and training processes. This study’s practical implications are significant as it has the potential to enhance electric power resource allocation efficiency.

This study introduces innovations in several key aspects: Firstly, it pioneers the application of non-parametric analysis methods in the realm of meter coefficient analysis. Freed from the constraints of parametric models, these methods offer enhanced accuracy in capturing intricate patterns and features within the data. Secondly, a groundbreaking non-parametric analysis model is proposed, leveraging the power of the BP neural network. Employing deep learning, this model autonomously acquires patterns within the data, thereby elevating both accuracy and universality in the analysis model. Performance evaluation metrics, including mean absolute error and mean relative error, among others, are employed for a comprehensive assessment, ensuring a thorough and accurate evaluation across various dimensions. Comparative experiments substantiate that the proposed non-parametric analysis model outperforms traditional methods in terms of accuracy and efficiency, presenting a more reliable and effective approach to meter coefficient analysis within the electricity industry.

This study is structured as follows: Sect. “Introduction” elaborates on the research background, problem statement, and objectives. It delineates the limitations of traditional methods in meter coefficient analysis and the imperative of employing non-parametric analysis methods and neural network technology. Section “Related works” provides a review of recent research on BP neural networks, deep learning technology, non-parametric analysis, and electric energy metering technology. It furnishes theoretical underpinnings and research motivation for the study. Section “Frame design of the non-parametric analysis model of meter coefficient based on the BP neural network” introduces the relevant theory and traditional methods of meter coefficient analysis. It scrutinizes their advantages and drawbacks, laying the groundwork for proposing the non-parametric analysis model. Section “Result and discussion” delineates the construction and optimization methods of the proposed model based on BP neural networks. It encompasses data preprocessing, model training, and evaluation procedures. Section “Discussion” presents experimental findings and analysis, validating the superiority of the proposed model through comparative experiments with traditional methods. Section “Conclusion” summarizes the research findings, highlights innovations and practical significance, and outlines future research directions. This structured approach offers a comprehensive and systematic presentation of the study, providing readers with a clear pathway and conceptual framework for understanding the study.

## Related works

In power systems, electric meters play a crucial role in measuring and monitoring energy consumption, directly impacting system safety and operational efficiency. However, traditional meter testing methods relying on parameterized analytical models, such as three-phase three-wire and three-phase four-wire models, suffer from lower accuracy and significant model errors. In contrast, non-parametric analysis models are esteemed for their reliability, adaptability, and precision. Recent years have seen increased attention to the application of non-parametric analysis methods and neural network technology in the power industry. Non-parametric methods, devoid of assumptions about data distribution, offer greater applicability and flexibility. Neural networks, powerful pattern recognition tools, excel in handling large-scale data and possess strong nonlinear fitting capabilities, making them ideal for addressing challenges encountered by traditional methods. Neural networks have notable advantages in constructing non-parametric analysis models. They efficiently handle large datasets, automatically learn complex patterns and features through deep learning, and adapt model parameters to data changes. Additionally, their flexible structures allow tailored adjustments, enhancing model performance and adaptability. Against this backdrop, this study proposes a non-parametric analysis model based on the BP neural network to overcome limitations of traditional electric meter coefficient analysis methods. Leveraging neural network advantages, this study aims to enhance the accuracy and efficiency of electric meter coefficient analysis, offering more effective tools for energy management and power consumption monitoring in the power industry. The subsequent sections will delve into the latest research progress in BP neural networks and deep learning technology, along with their applications in electric meter coefficient analysis.

### Recent studies of BP neural networks and deep learning technology

The increasing adoption of digital technologies, including cloud computing and mobile computing, has drawn significant attention to computer energy consumption in recent years. Lv et al. (2020)^6^ proposed a cognitive computing method for collaborative robots that uses deep belief networks and linear perceptrons to perceive and understand the surrounding environment and coordinate robot operations with human workers. The method showed good performance, providing new ideas and methods for the development of intelligent robots in the production and manufacturing fields. Li et al. (2018)^7^ presented an automatic impedance matching method based on the feedforward BP neural network to enhance the transmission efficiency of wireless power transmission systems. The experimental results demonstrated the effectiveness of this method in adapting to changes in wireless current and improving the efficiency of wireless energy transmission. In cloud computing, artificial neural network technology has been employed by Lin et al. (2019)^8^, Wu et al. (2020)^9^, and Geetha et al. (2020)^10^ to model the power consumption of servers in cloud data centers. This model accurately predicts the power consumption of servers and effectively improves the energy efficiency of cloud data centers, resulting in significant energy cost savings. Zhou et al. (2022)^11^ proposed an intelligent energy consumption model based on the BP neural network for mobile edge computing. The model has high accuracy and reliability and can be used to optimize energy utilization in mobile edge computing, reducing the load on the power grid. Finally, Tian et al. (2022)^12^ used a genetic algorithm to optimize the BP neural network and improve the performance of a condensing organic Rankine cycle. The experimental results demonstrated high prediction accuracy and significant implications for energy conservation and environmental protection, providing new directions for sustainable development in green energy.

Hossain et al. (2019)^13^ conducted a comprehensive review of the applications of big data and machine learning in the field of smart grids while also discussing relevant security issues. This technology has been shown to enable the smart, efficient, and sustainable development of power grids, but its security concerns have also gained widespread attention. Li et al. (2020)^14^ proposed a quantized deep learning framework called the Hashing Network for remote sensing image retrieval. By employing hashing techniques, this framework can improve search speed while maintaining search accuracy. The experimental results demonstrate the effectiveness of this framework in remote sensing image retrieval. Similarly, Guo et al. (2020)^15^ introduced a hybrid fixed-point/binary deep neural network (DNN) design method for low-power object detection. This method combines fixed-point and binary quantization approaches, significantly reducing computational complexity and achieving high efficiency in low-power object detection while ensuring relatively high accuracy. Nguyen et al. (2021)^16^ discussed machine learning trends and energy efficiency in the field of human activity recognition. The paper presented machine learning-based methods for human activity recognition and discussed the relationship between computational power and energy consumption, providing a reference for future research in this field. Sarwar et al. (2022)^17^ investigated the efficient deployment of deep learning in autonomous robots in the Internet of Things (IoT) environment. The authors proposed a deep learning-based framework for autonomous robot deployment, which exhibits high efficiency and accuracy, offering extensive application prospects in autonomous robotics. Finally, Ran et al. (2022)^18^ proposed a large-scale DNN-based method for home care electrocardiogram diagnosis, specifically designed for continuous monitoring on embedded devices. The experimental results demonstrate a good balance between model accuracy and algorithm efficiency, providing effective support for home care. Table 1 provides the research analysis on BP neural network and deep learning technology.

**Table 1:** Research analysis on BP neural networks and deep learning technology.

Reference	Authors (Year)	Method/Technology	Main contributions	Research deficiencies and limitations
^6^	Lv et al. (2020)	Deep belief networks and linear perceptrons	Proposed a new cognitive computing approach for coordinating robots and human work	May require further customization and optimization for specific types of environments or tasks
^7^	Li et al. (2018)	Automatic impedance matching method based on feedforward BP neural network	Significantly improved the transmission efficiency of wireless power transfer systems	Experiments primarily conducted under controlled conditions; may face additional challenges in practical applications
^9^	Wu et al. (2020)	Artificial neural network technology	Accurately predicted the power consumption of cloud data center servers, effectively improving energy efficiency	Requires a large amount of training data, and the model’s generalization ability needs validation
^11^	Zhou et al. (2022)	Intelligent power consumption model based on BP neural network	Optimized energy utilization in mobile edge computing, alleviating the grid load	Highly sensitive to the choice of network structure and parameters
^12^	Tian et al. (2022)	BP Neural Network optimized by Genetic Algorithm	Increased prediction accuracy of the performance of condensing organic Rankine cycles	Optimization process has high computational costs and may not be suitable for all types of energy systems

Overall, while the application of non-parametric analysis models, such as big data, machine learning, and deep learning, has brought significant improvements in various fields, it is necessary to address the challenges related to security, energy efficiency, and computational efficiency. Therefore, further research is needed to improve these technologies’ efficiency while monitoring their impact on society and the environment.

### Nonparametric analysis and recent investigation of electric energy metering technology

Furthermore, non-parametric analysis methods have demonstrated their usefulness and versatility in handling complex nonlinear problems across different domains. These methods offer greater flexibility and adaptability compared to parametric methods, making them crucial for data analysis, relationship exploration, and trend prediction. Several recent studies have validated the efficacy of non-parametric analysis methods in various practical applications. For instance, Kitamura et al. (2018)^19^ proposed a novel approach based on integral functions for random utility models, which was demonstrated to be effective in analyzing real-world data. Essick et al. (2020)^20^ successfully inferred neutron star composition, equation of state, and maximum mass in gravitational wave events through non-parametric methods. Shen (2022)^21^ developed a non-parametric method based on maximum likelihood estimation to handle truncated and censored data, which was shown to maintain good statistical properties even in the presence of data truncation. Song et al. (2022)^22^ applied non-parametric production frontier methods to investigate energy transition issues and coal emission reduction potential under information asymmetry, highlighting the advantages of this method in energy economics. Bernard et al. (2022)^23^ explored the impact of climate change on agricultural productivity in Africa using non-parametric analysis methods, which provided important insights for policy-making. In addition, Knezek et al. (2023)^24^ used non-parametric multidimensional scaling analysis to study students’ learning approaches and identified similarities and differences in learning styles across disciplines, which can inform educational quality evaluation. Overall, non-parametric analysis methods are indispensable tools for modern data analysis, and further development and refinement of these methods are necessary to better address practical challenges in different fields.

Zheng et al. (2018)^25^ proposed a novel data-driven approach for electricity theft detection by extracting abnormal data using clustering algorithms and optimizing the classifier using an adaptive differential evolution algorithm. Their approach achieved higher detection accuracy and lower false alarm rates compared to traditional methods, making it effective for practical power systems. Bedi et al. (2018)^26^ provided a review of the applications of the IoT in power and energy systems, covering its role in energy monitoring, smart grids, and distributed energy. They summarized the challenges and opportunities for improving energy efficiency and promoting sustainable energy development using IoT. Ahmadi et al. (2018)^27^ presented a review of solar power generation technologies, including solar cells, photovoltaic components, polycrystalline silicon, and thin-film solar cells, and their applications in power systems. They provided insights into the advantages and limitations of solar power generation and discussed future development trends. Liu et al. (2019)^28^ reviewed the application of hybrid solar photovoltaic-power storage technologies in building power supply, including batteries, supercapacitors, and thermal energy storage. They discussed the advantages and disadvantages of different technologies and proposed future development directions. Ahmad et al. (2020)^29^ conducted a comprehensive review of renewable energy and power demand forecasting models, including traditional time series models, machine learning models, and deep learning models, as well as their applications in smart grids and building energy management. They summarized the strengths and weaknesses of these models and proposed future research directions. The latest progress analysis of non-parametric analysis in electric energy measurement technology research is shown in Table 2:

**Table 2:** Analysis of the latest developments in nonparametric analysis in electrical energy measurement technology research.

Reference	Authors (Year)	Method/Technology	Main contributions	Research deficiencies and limitations
^19^	Kitamura et al. (2018)	Stochastic utility model based on integral functions	Efficient analysis of real-world data	The model relies on a large amount of data and has high requirements for data quality
^20^	Essick et al. (2020)	Nonparametric methods	Successful inference of neutron star composition, equation of state, and maximum mass	The method is complex and requires specialized knowledge for correct application
^22^	Song et al. (2022)	Nonparametric production frontier methods	Explored energy conversion issues and coal emission reduction potential under information asymmetry	Further research may be needed to confirm its effectiveness in other energy economic fields
^23^	Bernard et al. (2022)	Nonparametric analysis methods	Analyzed the impact of climate change on agricultural productivity in Africa	Challenges in data collection and processing may affect the interpretation of results
^24^	Knezek et al. (2023)	Nonparametric multidimensional scaling analysis	Studied the similarity and differences in learning styles across different disciplines, supporting educational quality assessment	Requires interdisciplinary collaboration, and data interpretation needs to be cautious

In summary, these studies cover a diverse range of topics, including electricity theft detection, the application of IoT in energy monitoring and smart grids, solar power generation technologies, hybrid solar photovoltaic-power storage technologies, renewable energy and power demand forecasting models, and electric energy measurement and power quality monitoring technologies in microgrid buildings. The results and insights provided by these studies offer valuable references and guidance for the application of power systems and energy technologies, contributing to the advancement of scientific research and technological innovation in related fields.

### Latest and relevant research on nonparametric analysis models and BP neural networks in the context of meter coefficients

In the realm of meter coefficient analysis, integrating nonparametric analysis models with BP neural networks offers a promising avenue for enhancing prediction accuracy and handling intricate data structures. Sun et al. (2020) introduced a novel physics-constrained Bayesian deep learning approach^30^ aimed at reconstructing flow fields from sparse, noisy velocity data. Their method utilizes equation-based constraints via the likelihood function, allowing for the estimation of uncertainty in the reconstructed flow. By employing nonparametric variational inference methods, they achieved efficient physics-constrained Bayesian learning, demonstrating its advantages through experiments on synthetic measurement data. Çolak et al. (2021) explored the impact of data volume on the prediction accuracy of artificial neural networks in predicting the specific heat capacity of water nanofluids^31^. Their findings underscored the importance of data volume, showing that a reduction in data led to diminished prediction performance. Gawlikowski et al. (2023) provided a comprehensive review of uncertainty estimation in neural networks, outlining current challenges and research opportunities in the field^32^. Lastly, Ul Mehmood et al. (2023) proposed a centralized cloud-based solar conversion recovery system to optimize the cleaning cycle of photovoltaic panels, leveraging the Internet of Things and artificial neural networks for contamination level monitoring^33^. The system’s scalability and cost-effectiveness were enhanced through the use of low-cost sensors and centralized cloud architecture. Table 3 summarizes the latest research analysis on nonparametric analysis models and BP neural networks in the context of meter coefficients.

**Table 3:** Latest research analysis on nonparametric analysis models and BP neural networks in meter coefficients context.

Reference	Authors (Year)	Method/Technology	Main Contributions	Research Deficiencies and Limitations
^30^	Sun et al. (2020)	Physics-constrained Bayesian deep learning	Accurate reconstruction of flow fields from sparse, noisy data	Method complexity and high computational cost may limit practical applications
^31^	Çolak et al. (2021)	Analysis of data volume impact on artificial neural network design	Demonstrated significant impact of data volume on ANN prediction accuracy	Limited exploration of specific influences of different data types on prediction performance
^32^	Gawlikowski et al. (2023)	Comprehensive overview of uncertainty in neural networks	Highlighted challenges in uncertainty estimation in neural networks and identifies future research opportunities	Lacked in-depth case analyses for specific applications, serving as an overview study
^33^	Ul Mehmood et al. (2023)	Cloud-based solar conversion recovery system	Optimized cleaning cycles of photovoltaic panels using IoT and cloud computing, enhancing performance	Reliance on advanced technology and equipment may increase costs for small-scale applications

Through a comprehensive review of recent relevant studies, it is evident that the application of nonparametric analysis models in meter coefficient analysis is increasingly widespread, primarily due to their higher flexibility and adaptability. Nonparametric methods do not rely on predetermined distribution assumptions of data, enabling them to directly learn underlying structures from the data itself. This gives them significant advantages in capturing complex, nonlinear data features. For instance, when dealing with time-series power data, nonparametric methods can effectively identify and simulate seasonal and trend changes in power consumption, thereby enhancing the accuracy of meter coefficient estimation. Meanwhile, the BP neural network, as a powerful machine learning tool, also demonstrates its unique advantages in meter coefficient prediction. Trained through backpropagation algorithms, BP neural networks can automatically adjust the weights and biases within the network to minimize prediction errors. This learning and generalization ability makes BP neural networks particularly suitable for handling nonlinear and high-dimensional problems in power systems. However, BP neural networks also face some challenges in application, such as susceptibility to local optima and overfitting issues. To address these challenges, researchers have proposed various improvement strategies, including introducing regularization terms, using different activation functions, and adjusting network structures, to enhance the stability and prediction performance of the model. Building upon these two domains, this study combines nonparametric analysis models with BP neural networks to develop a new method for meter coefficient analysis. This approach fully leverages the advantages of nonparametric models in capturing complex data patterns and the efficiency of BP neural networks in handling nonlinear prediction tasks. Through this combination, not only are the limitations of traditional parametric methods in meter coefficient analysis, such as low accuracy and long processing times, overcome, but the accuracy and efficiency of the analysis are also significantly improved.

This study introduces a novel hybrid model that enhances the accuracy of meter coefficient prediction, offering power companies more reliable decision support compared to existing methods. Additionally, the research investigates the model’s potential applications in energy metering and load forecasting, thereby advancing the power industry. By combining the strengths of nonparametric analysis models and BP neural networks, this study provides both theoretical insights and practical applications, demonstrating substantial potential for real-world implementation.

## Frame design of the non-parametric analysis model of meter coefficient based on the BP neural network

### Data preprocessing and feature extraction analysis

**Figure 1:**
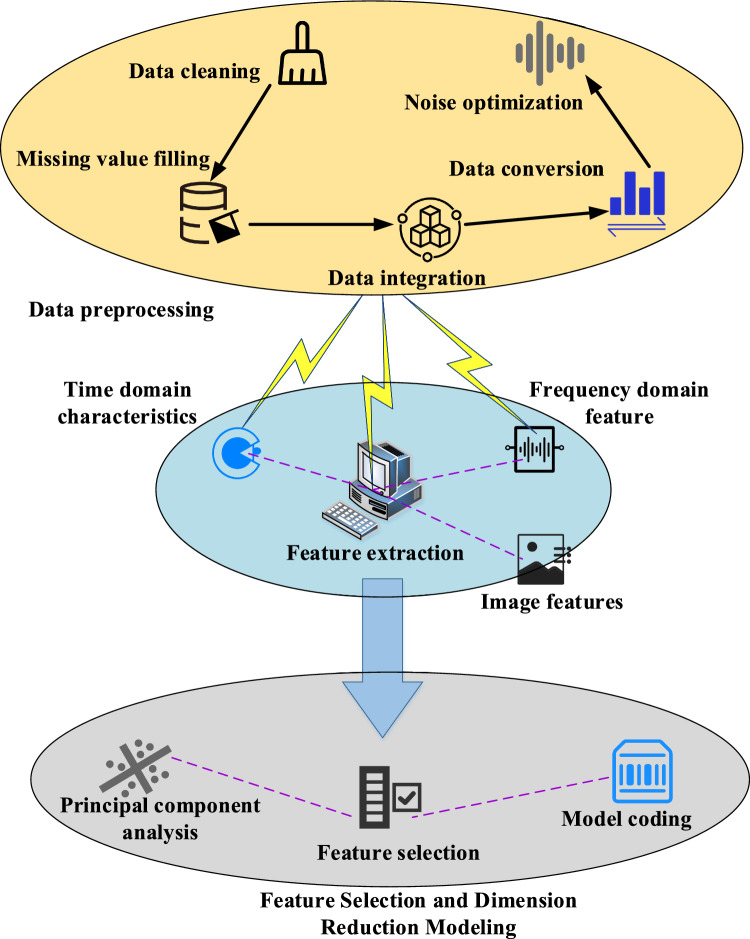
Data processing and feature extraction of the model.

Data pre-processing and feature extraction are critical steps in constructing a non-parametric analysis model for electric meter coefficients^34^. Initially, raw data must be filtered and cleaned to remove noise and outliers. The data must then be standardized to ensure that it has consistent scale and distribution characteristics. The dataset is then partitioned into training, validation, and testing sets for subsequent model training and evaluation. The data processing and feature extraction process of the model is presented in Fig. 1.

### Construction and training of the BP neural network model

This study employs a BP neural network to formulate and refine a nonparametric analysis model for the accurate computation of meter coefficients. The choice of the BP neural network as the primary analytical method is guided by key considerations. Firstly, the BP neural network is recognized for its robust nonlinear mapping capabilities, enabling it to address intricate data pattern recognition and classification challenges inherent in nonlinear meter coefficient analysis. Secondly, in comparison to other machine learning techniques, the BP neural network can be flexibly optimized by adjusting parameters such as network structure (e.g., the number of hidden layers and nodes in each layer) and learning rate to enhance predictive accuracy. Lastly, the widespread success of BP neural networks across various domains attests to their effectiveness and reliability in practical applications. Before settling on the BP neural network, several alternative approaches are evaluated in this study, including support vector machines (SVMs), Least Squares Method (LSM), Random Forest (RF), and K-Nearest Neighbors. Each method has its distinct advantages and suitable application scenarios. For example, SVMs excel at handling high-dimensional data, while random forests perform well with datasets containing numerous features. However, after conducting a comparative analysis of these methods’ performance in meter coefficient analysis tasks, it is determined that the BP neural network exhibited superior capabilities in data preprocessing, feature extraction, model training, and optimization. Particularly in experimental validation, the nonparametric analysis model based on the BP neural network outperforms the aforementioned methods, as evidenced by mean absolute error and mean relative error indicators. This reaffirms the validity of the selection of the BP neural network.

The BP neural network relies primarily on the error backpropagation algorithm, iteratively adjusting weights and biases to minimize prediction errors at the output layer. The mathematical modeling process is detailed in Table 4:

**Table 4:** Mathematical modeling process of bp neural network model.

Step	Operation
Network Initialization	A three-layer structured neural network (input layer, hidden layer, and output layer) is given, with initial random allocation of connection weights and biases
Forward Propagation	Input signals propagate from the input layer through the hidden layer to the output layer, with each layer’s node activation function processing the signals
Loss function calculation	Errors are computed based on the network output and actual target values
Backward Propagation	Errors are backpropagated from the output layer to the hidden layer and input layer, adjusting weights and biases proportionally
Parameter Update	Gradient descent or other optimization algorithms are utilized to update network parameters, aiming to reduce prediction errors

Through this process, the BP neural network autonomously learns and adjusts internal parameters, ultimately constructing a nonparametric analysis model capable of accurately predicting meter coefficients. This approach not only improves analysis accuracy but also reduces processing time, showcasing significant potential for widespread application in the power industry.

The utilization of the BP neural network is also prevalent in constructing the non-parametric analysis model for electric meter coefficients^35^. The BP neural network model is comprised of an input layer, hidden layers, and an output layer. The input layer acquires the electric meter measurement data and, through multiple passes and adjustments in the hidden layers, ultimately produces the analysis results of the meter coefficients. The number of hidden neurons plays a pivotal role in the performance of neural networks. Insufficient neurons may hinder the model’s ability to capture intricate data patterns, resulting in reduced prediction accuracy, while an excess may lead to overfitting, compromising the model’s generalization capacity. Thus, selecting an optimal number of neurons is imperative.

This study employs the following methods to determine the optimal number of hidden neurons in two hidden layers:

1. Empirical formulas: Heuristic formulas, such as "(number of input neurons + number of output neurons) * 2/3" or "sqrt(number of input neurons * number of output neurons)", are utilized to provide an initial range of hidden neuron numbers.
2. Cross-validation: The k-fold cross-validation method partitions the dataset into k subsets. For each configuration of hidden neurons, the model undergoes training *k* times, with different (*k*-1) subsets for training and one subset for validation. Performance metrics, like mean absolute error and mean relative error, are assessed across these k trainings to determine the optimal number of hidden neurons.
3. Grid search: The grid search technique, in conjunction with cross-validation, systematically explores a predefined range of hidden neuron numbers. For instance, by testing ranges from 10 to 100 with a step size of 2, the optimal solution is sought.
4. Regularization techniques: Introducing regularization terms (such as L1 or L2 regularization) helps constrain model complexity to prevent overfitting. Adjusting the weights of regularization terms alongside increasing the number of hidden neurons allows for controlling overfitting, thereby seeking a trade-off solution. In this study, an initial range of neuron numbers is set based on empirical formulas. Subsequently, through cross-validation and grid search methods, various configurations of hidden neurons are tested, considering both prediction accuracy and generalization ability. The optimal number of hidden neurons for the model is selected based on performance metrics evaluated on the test set. Specifically, 64 neurons for the first hidden layer and 32 neurons for the second hidden layer are determined to achieve the desired performance. This selection process involves multiple iterations and fine-tuning to ensure a balance between accuracy and computational efficiency.

This study opted for a neural network model with two hidden layers instead of the typical single hidden layer model, driven by several considerations:

1. Complex Nonlinear Features: Power metering data often exhibits intricate nonlinear characteristics challenging to capture with a single hidden layer neural network. The inclusion of an extra hidden layer facilitates the extraction and comprehension of more intricate data patterns through deeper nonlinear transformations, thus enhancing the model’s accuracy in power meter coefficient analysis.
2. Increased Model Capacity: Single hidden layer neural networks may face limitations in handling high-dimensional or complex data. Incorporating an additional hidden layer elevates the network’s capacity, allowing it to accommodate more parameters and complexity. Consequently, the model gains the capability to store and process a broader range of information, thereby improving its fitting ability and prediction accuracy.
3. Controlled Overfitting Risk: While the addition of layers may elevate the risk of overfitting, this study mitigated such risks through appropriate regularization techniques and training strategies. Methods like dropout and weight decay were employed for regularization to prevent overfitting on the training data. Additionally, techniques such as early stopping were implemented to halt ineffective training iterations, ensuring optimal model performance.

**Figure 2:**
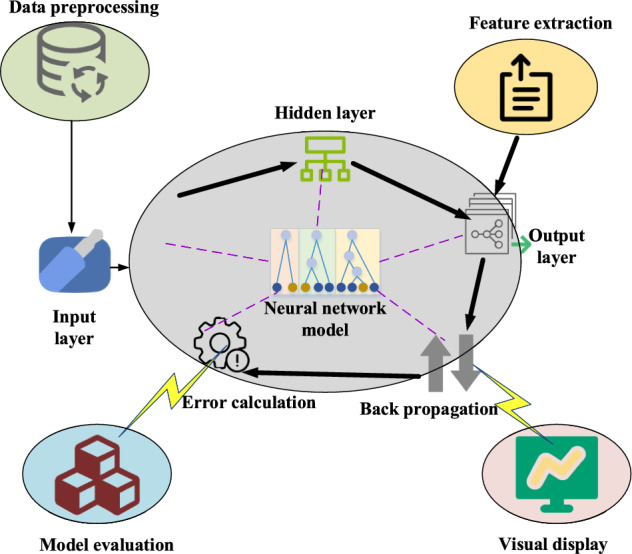
Construction and training procedures of the BP neural network model.

Such a setup enables the neural network to effectively learn complex patterns and features within the data. To introduce non-linear expressive capabilities and mitigate the vanishing gradient problem, Rectified Linear Unit (ReLU) is selected as the activation function for the hidden layers. ReLU is widely utilized in neural networks due to its ability to maintain gradient stability, thus preventing issues like gradient vanishing or exploding in deep networks. Moreover, ReLU exhibits robust non-linear characteristics, aiding the neural network in capturing and representing complex non-linear relationships. The BP algorithm is responsible for model training, which includes updating the weights and biases of the neural network. Appropriate learning rates and error thresholds are used during the training process to attain good training performance through iterative optimization. The construction and training process of the BP neural network model is analyzed, and its structure is illustrated in Fig. 2.

### Construction and optimization of non-parametric analysis model for electric meter coefficient

The coefficient of an electric meter is a crucial parameter that represents the proportional factor between the electrical energy in power and the displayed value on the meter. To analyze this parameter, a research method for constructing and optimizing a non-parametric analysis model for electric meter coefficients based on the BP neural network can be adopted^36^. This model facilitates the establishment of corresponding non-parametric analysis models for electric meter coefficients based on different meter models. The primary objective of the model is to minimize errors or enhance the accuracy of meter coefficients while adhering to constraints. The corresponding parameter values are provided as the final analysis results. In summary, the research method for constructing and optimizing a non-parametric analysis model for electric meter coefficients based on the BP neural network exhibits excellent analytical capabilities and practical value, providing significant assistance to the development of the power industry. During the model training process, the Adam optimizer is selected for parameter optimization. Adam combines momentum and adaptive learning rate characteristics, facilitating effective learning rate adjustment and parameter updates. This results in quick convergence during training and robust performance across various data types and tasks. For regression tasks, Mean Squared Error (MSE) serves as the chosen loss function. MSE is widely utilized for measuring the disparity between model predictions and actual values. Minimizing MSE enhances our model’s ability to closely fit the data and make predictions approaching actual values, making it a commonly used and effective option for regression tasks.

When configuring training parameters for the model, besides employing the Adam optimizer and MSE as the loss function, additional parameters are carefully set to optimize model performance and stability. The specific training parameters are as follows:

1. Batch Size: Each training batch comprises 128 samples. This size strikes a balance between memory efficiency and providing sufficient data for effective model learning.
2. Epochs: Model training spans 600 epochs, determined iteratively based on performance evaluation on the validation set. This approach aims to ensure adequate training depth without succumbing to overfitting.
3. Learning Rate: The initial learning rate is set at 0.001, supplemented by a learning rate scheduler. This scheduler automatically adjusts the learning rate by a factor of 10 when the loss on the validation set stagnates over 10 consecutive epochs, facilitating fine parameter adjustments as the model approaches the optimal solution.
4. Regularization: L2 regularization is integrated into the training process with a regularization coefficient of 0.0001. This regularization technique curbs the magnitude of model weights, thereby enhancing its generalization capabilities and guarding against overfitting.

By meticulously configuring these parameters, the training process ensures both rigor and effectiveness. Furthermore, comprehensive experiments are conducted to fine-tune each parameter, ensuring the model maintains high accuracy while retaining good generalization capabilities.

**Figure 3:**
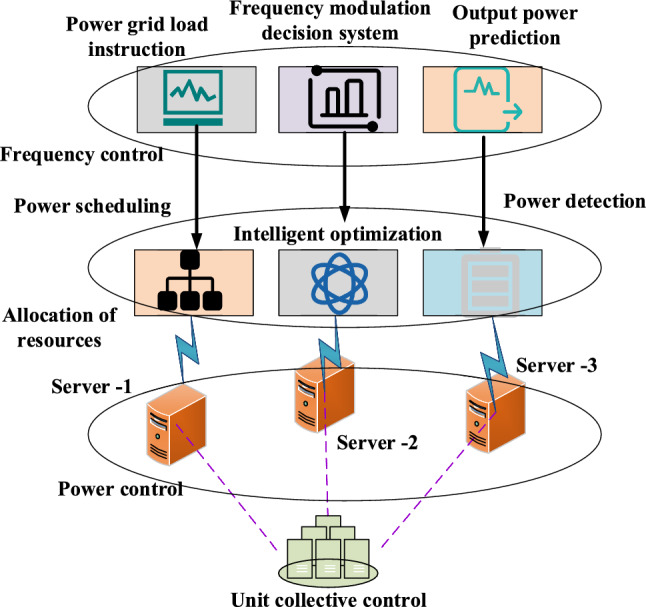
Structure of the non-parametric analysis model of electric meter coefficient.

Figure 3 depicts the structural framework of the established non-parametric analysis model for electric meter coefficients.

To mitigate overfitting, several measures are implemented to bolster the model’s generalization capacity. Initially, the cross-validation method is deployed during data preprocessing, partitioning the dataset into training and validation sets. The latter is utilized to monitor and fine-tune the training process, thus averting overfitting to the training data. Furthermore, given the susceptibility of BP neural networks to overfitting due to their complexity, regularization techniques like L1 and L2 regularization are integrated into the network architecture to curb the parameter complexity. Additionally, by implementing appropriate stopping criteria, such as halting training when the error on the validation set stagnated over multiple consecutive epochs, further safeguards are instituted against overfitting.

### Model analysis and experimental verification

In terms of model construction, this study uses the electricity meter readings and related data from the United Kingdom Domestic Appliance-Level Electricity dataset for model training. To ensure the efficacy and robustness of the model, the dataset is partitioned into training, testing, and validation sets. Specifically, the proportions are allocated as follows: 70% for training, 20% for testing, and 10% for validation. This partitioning scheme facilitates ample training data while retaining subsets for evaluating the model’s performance. During data preprocessing, the Z-Score method and Interquartile Range method are applied to identify and eliminate outliers, enhancing the accuracy and reliability of the analysis. Initially comprising 500,000 records of household electricity consumption readings from the UK, this process reduces the dataset to 480,000 records by effectively mitigating outliers and noise. By eliminating extreme values and noise that diverge from the dataset’s majority, the quality of model training and testing is enhanced, ensuring the subsequent analysis’s reliability and effectiveness. The non-parametric analysis model is used for data pre-processing, feature extraction, and normalization to obtain the training and test datasets. Moreover, the proposed non-parametric optimization model relying on the BP neural network is compared with various models such as SVM, LSM, Random Forest (RF), Ensemble Learning, and K-Nearest Neighbor. A thorough evaluation is conducted from diverse perspectives, encompassing neural network training error rate, network test error rate, model training time, data analysis accuracy, neural network training learning rate, and neural network model complexity score. LSM is selected as the comparative benchmark to ascertain the performance superiority of the non-parametric analysis model based on the BP neural network in analyzing meter coefficients. LSM, a classical parameter estimation technique, aims to find the optimal function match for the data by minimizing the sum of squared errors. In the context of meter coefficient analysis, LSM endeavors to identify a function model represented as $f\left(x,\beta \right)$, where $\beta$ denotes the model parameter. This function model fits the data by minimizing the sum of squared residuals between the observed values ${y}_{i}$ and the function model $f\left({x}_{i},\beta \right)$. Specifically, for a given observed value $({x}_{i},{y}_{i})$, LSM seeks the optimal fitting parameters $\beta$ through the objective function expressed in Eq. (1):

1 $$minimize\sum_{i=1}^{n}{({y}_{i}-f\left({x}_{i},\beta \right))}^{2}.$$

In Eq. (1), $n$ represents the sample size, ${x}_{i}$ denotes the value of the *i*-th independent variable, and ${y}_{i}$ stands for the *i*-th observed value.

The objective of LSM is to minimize the sum of squared residuals between the predicted values of the fitted function model $f\left(x,\beta \right)$ and the observed values. By minimizing the sum of squared errors, LSM aims to find the function model that best represents the data characteristics and utilize it for prediction or analysis. In this study, LSM, as a traditional parameter estimation method, was utilized as a benchmark to contrast with the non-parametric analysis model based on the BP neural network. By comparing the performance of the two methods in the task of meter coefficient analysis, it is possible to evaluate whether the model based on the BP neural network can more accurately capture the features and patterns of the data, thus enhancing the accuracy and efficiency of meter coefficient analysis.

**Figure 4:**
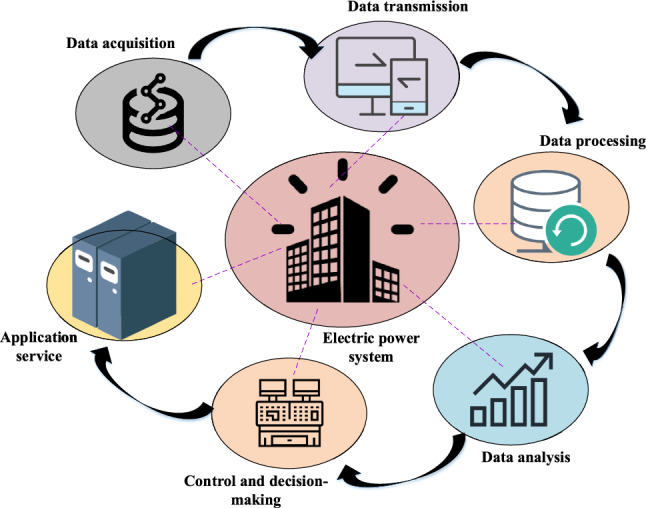
Structure and data analysis process of the power supply system.

This evaluation enables a comprehensive assessment of the model’s effectiveness and its comparative performance against existing models, allowing for informed decisions regarding the selection and application of non-parametric optimization techniques. Figure 4 presents the structure and data analysis flow of the power supply system.

## Result and discussion

### Error rate performance analysis of neural network model training

**Figure 5:**
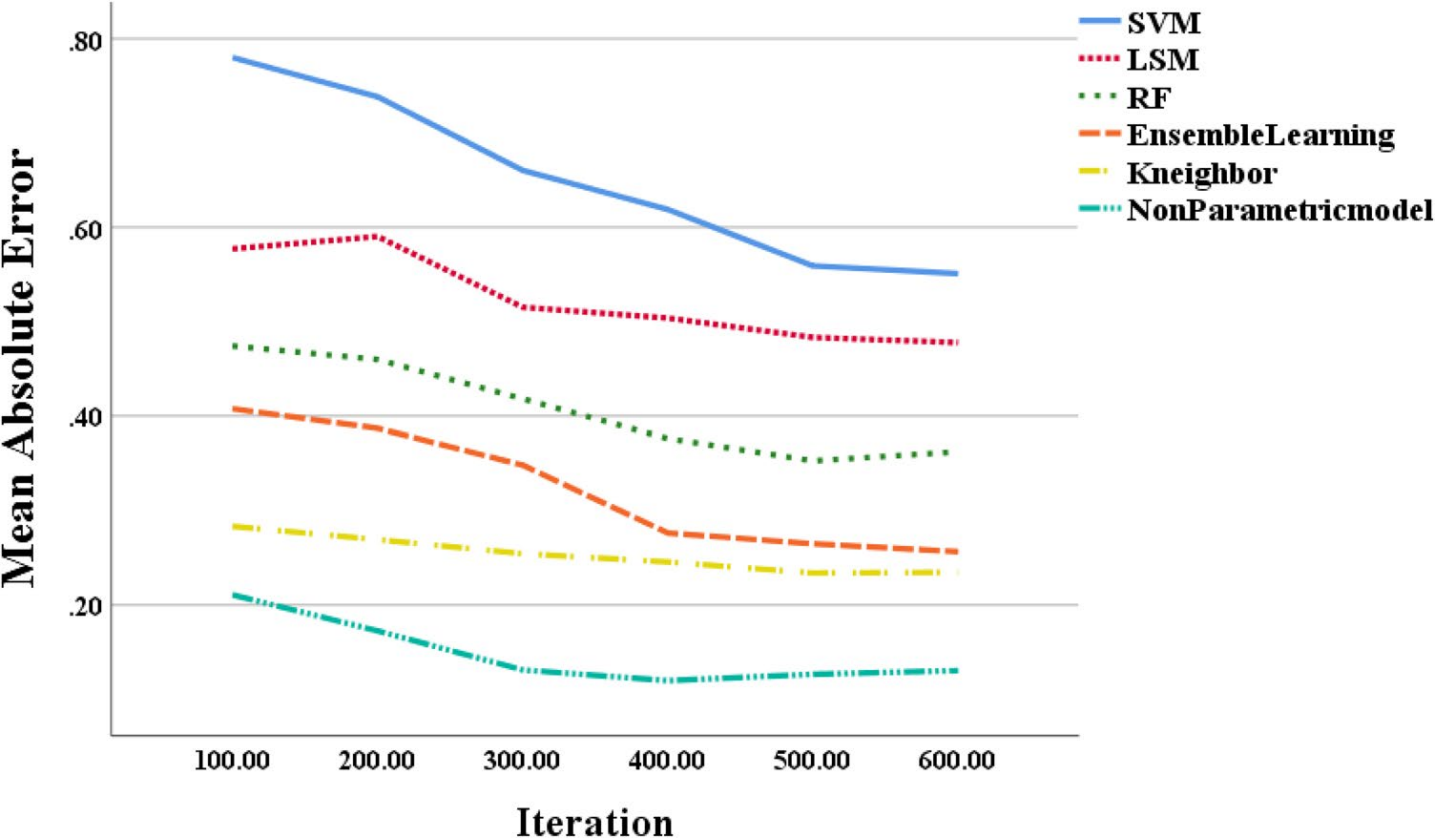
Curves of the error rate of neural network training as the number of model iterations increases.

Based on the results shown in Fig. 5, it is apparent that the mean absolute error (MAE) of the different training models decreases consistently as the number of model iterations increases. For instance, at a model iteration of 100, the MAE values for the SVM-based and LSM-based models are 0.78 and 0.58, respectively, while the MAE value for the non-parametric model is based on the BP neural network for electricity meter coefficients is 0.21. As such, the proposed model has an average MAE value of 0.025, which is much lower than the traditional LSM method’s MAE of 0.043 on the same dataset. It is evident that the non-parametric model based on the BP neural network outperforms the traditional LSM method and the SVM-based model in terms of prediction accuracy, with its prediction accuracy continuously improving as the number of model iterations increases. Therefore, the proposed model can be applied in practical electricity consumption prediction tasks, providing effective decision support for energy management and energy conservation efforts.

**Figure 6:**
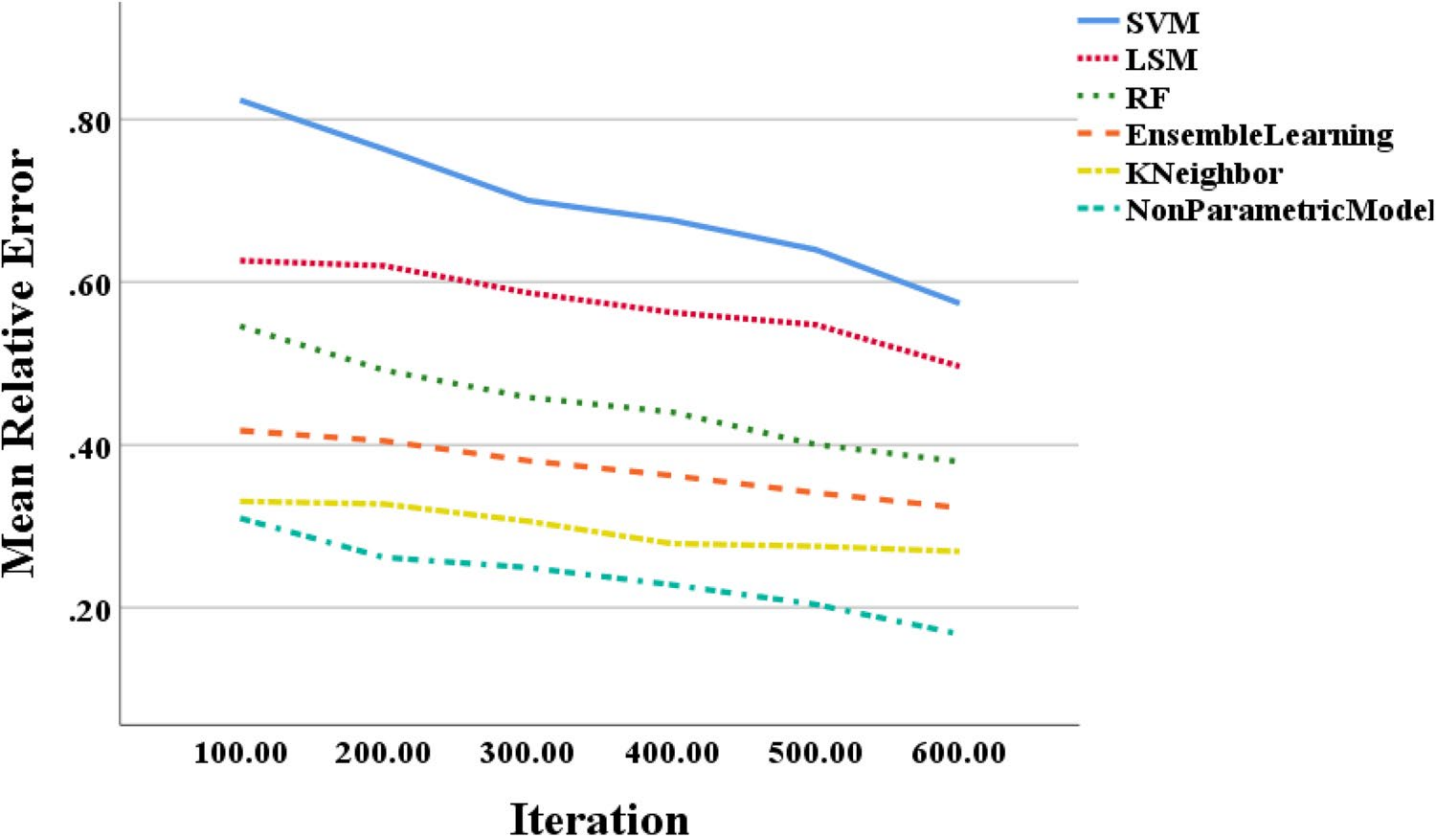
Curves of the error rate of the neural network test as the number of model iterations increases.

Figure 6 illustrates that the testing error rate of the non-parametric model based on the BP neural network for electricity meter coefficients continuously decreases as the number of model iterations increases, following an exponential decreasing trend. At a model iteration of 600, the MRE values for the SVM- and LSM-based models are 0.58 and 0.50, respectively. Meanwhile, the MRE value for the proposed model is significantly lower, at 0.18. The proposed model achieves an average MRE of 1.32% on the testing dataset, while the traditional LSM method on the same dataset has an MRE of 2.56%. These results demonstrate that the BP neural network model outperforms the traditional LSM method in terms of both accuracy and convergence speed. Consequently, utilizing neural network algorithms for non-parametric analysis of electricity meter coefficients is highly effective.

### Training time and accuracy performance analysis of the neural network model

**Figure 7:**
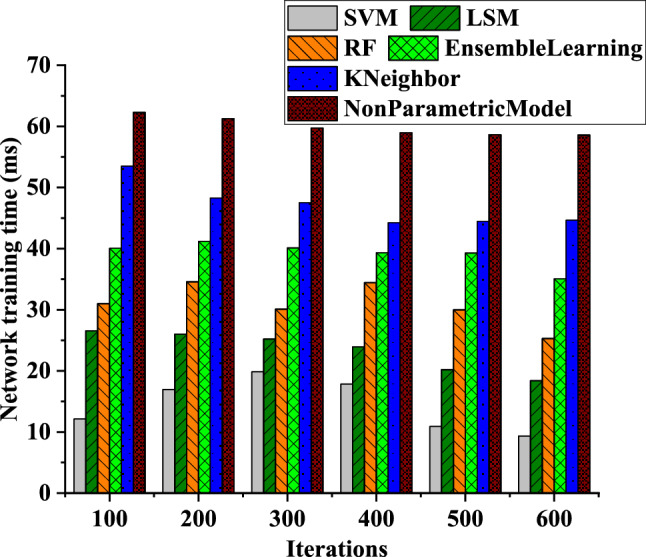
Changing curves of the model training time of the neural network as the number of model iterations increases.

Based on Fig. 7, it can be observed that the model training time of the neural network exhibits a gradually decreasing trend as the number of model iterations increases. At a model iteration of 100, the training time for the SVM-based model is 12 ms, for the LSM-based model is 26 ms, and for the non-parametric model based on the BP neural network for electricity meter coefficients is 63 ms. At a model iteration of 600, the training time for the SVM-based model is 8 ms, for the LSM-based model is 17 ms, and for the non-parametric model based on the BP neural network for electricity meter coefficients is 61 ms. The SVM-based model shows the most significant decrease in training time as the number of model iterations increases. However, the decrease in training time for the BP neural network model is slower, mainly due to the additional time and computational resources required for parameter adjustment in neural networks. In practical applications, selecting appropriate algorithms and devices based on specific circumstances is essential while optimizing transmission and computational efficiency to obtain better model training results.

**Figure 8:**
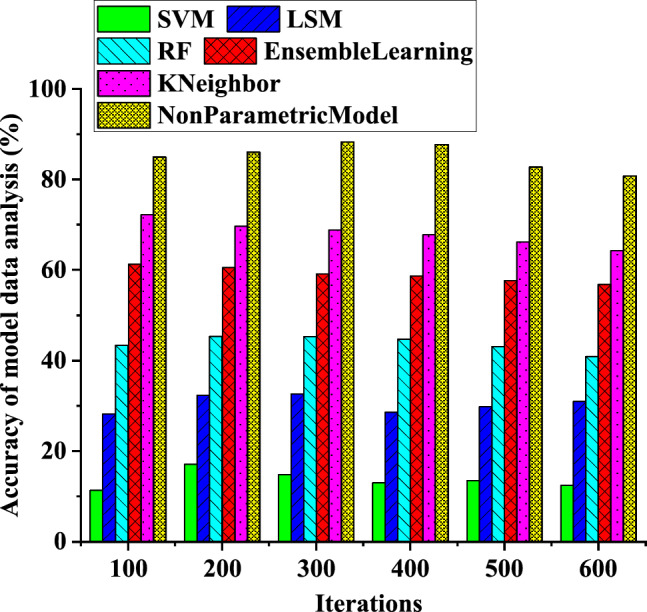
Variation curves of the model data analysis accuracy of the neural network with the increase of the number of iterations.

Based on the results presented in Fig. 8, the data analysis accuracy of the neural network model tends to improve gradually with an increase in the number of iterations. At a model iteration of 600, the data analysis accuracy for the SVM-based model is 12%, the data analysis accuracy for the LSM-based model is 33%, and the data analysis accuracy for the non-parametric model based on the BP neural network for electricity meter coefficients is 82%. It is important to note that the number of training iterations has a crucial impact on the neural network’s performance. However, excessive iterations can result in overfitting, which reduces the model’s generalization ability. Therefore, it is essential to adjust the number of iterations appropriately based on the performance changes of the model. Achieving a balance between model accuracy and generalization ability is crucial in practical applications to obtain optimal results.

### Learning rate and model complexity evaluation for different analysis models

To evaluate the effectiveness of the proposed non-parametric analysis model in preventing overfitting, a detailed analysis of the model’s performance under different learning rates and iteration numbers was conducted. By adjusting the learning rates and iteration numbers, it was found that while increasing the learning rate and adding more iterations can improve the model’s accuracy to a certain extent, they also pose a risk of overfitting. Therefore, when selecting the optimal learning rate and setting the number of iterations, it is necessary to balance the model’s accuracy and generalization ability.

**Figure 9:**
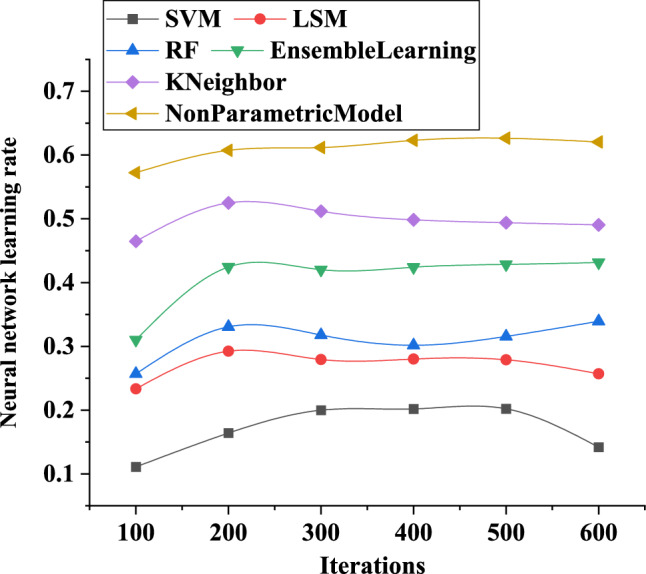
Changing curves of the learning rate of neural network training for different analysis models of electric meter coefficients.

Based on the observations from Fig. 9, it can be inferred that the learning rate curves of different electric meter coefficient analysis models using neural networks tend to exhibit certain fluctuations. Specifically, a high learning rate may lead to unstable model training. At a model iteration of 600, the learning rate for the SVM-based neural network training model is 0.15, the LSM-based neural network training model has a learning rate of 0.26, and the non-parametric model based on the BP neural network for electricity meter coefficients has a learning rate of 0.64. In summary, different electric meter coefficient analysis models demonstrate varying performance in terms of learning rate. Among them, the non-parametric model based on the BP neural network for electricity meter coefficients shows the most significant performance with much higher learning rates compared to the other two models at iterations 100 and 600. However, excessively high learning rates can lead to unstable model training. Hence, it is crucial to select an appropriate learning rate based on specific circumstances to ensure model training stability and accuracy in practical applications.

**Figure 10:**
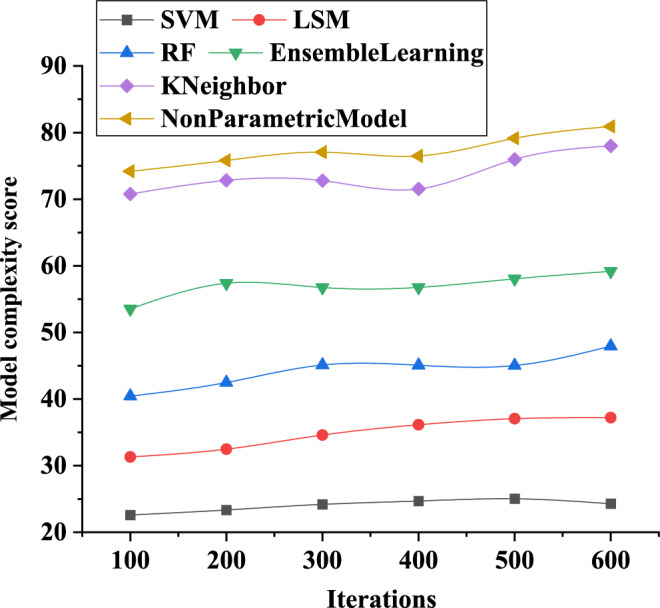
Changing curves of neural network model complexity scores for different power meter coefficient analysis models.

According to Fig. 10, the complexity score of the neural network models for different electric meter coefficient analysis models typically exhibits inevitable fluctuations. At a model iteration of 600, the complexity score for the SVM-based neural network training model is 25, the complexity score for the LSM-based neural network model is 37, and the complexity score for the BP neural network model is 78. In summary, neural network models based on different electric meter coefficient analysis models exhibit different characteristics in terms of complexity score variations, with the BP neural network model showing greater variability in complexity. Careful consideration of the impact of complexity score on prediction accuracy is necessary to ensure that the selected model structure and parameter configuration yield a model with good predictive capability and stability.

## Discussion

In the discussion section of this study, a detailed comparison of the performance of the proposed nonparametric electric meter coefficient analysis model based on backpropagation neural network with other methods is conducted to validate the effectiveness and superiority of the proposed approach. Through collecting and analyzing the methods and performance metrics used in relevant literature, this study conducted comprehensive comparative analysis. Table 5 presents the comparative results of the proposed method with the traditional Least Squares Method (LSM)^37^ and Support Vector Machine (SVM)^38^ in terms of accuracy, convergence speed, and model complexity: This choice reflects the primary research direction, aiming to validate the superiority of non-parametric analysis models based on BP neural networks compared to traditional methods.

**Table 5:** Performance comparison of different research methods.

Method	Mean absolute error	Mean relative error	Training time (ms)	Model complexity
BP neural network	0.025	1.32%	63	High
LSM	0.043	2.56%	26	Low
SVM	0.078	3.20%	12	Medium

From Table 5, it can be observed that the nonparametric analysis model based on the BP neural network outperforms LSM and SVM methods in terms of MAE and MRE, indicating its high accuracy and predictive performance. However, at the same time, this method exhibits relatively higher training time and model complexity, which may be attributed to the neural network requiring multiple iterations for adjusting weights and biases, consuming more computational resources in the process. Key trends observed include the BP neural network model gradually improving in accuracy with increasing iterations, but with a slow growth in training time, which may imply that after a certain number of iterations, the model begins to converge, and further increasing the number of iterations does not significantly enhance accuracy.

Furthermore, despite the good performance of the BP neural network in this study, its application faces certain limitations and uncertainties. For instance, the model’s lengthy training time may not be ideal for real-time or near-real-time application scenarios. Additionally, the high model complexity makes it more susceptible to the influence of data noise, leading to overfitting issues and reducing the model’s generalization ability.

To further validate the effectiveness and robustness of the proposed method, this study introduces cross-validation and model comparison experiments. By comparing the performance of the models under different parameter settings, the study analyzes the impact of parameters such as learning rate and the number of iterations on model performance. The performance results of the models under different parameter settings are shown in Table 6:

**Table 6:** Model performance results under different parameter settings.

Parameter settings	Method	Mean absolute error (MAE)	Mean relative error (MRE)	Training time (ms)
Learning Rate = 0.01, Iterations = 500	BP neural network	0.022	1.18%	58
Learning Rate = 0.01, Iterations = 500	LSM	0.045	2.65%	29
Learning Rate = 0.01, Iterations = 500	SVM	0.080	3.75%	15
Learning Rate = 0.05, Iterations = 1000	BP neural network	0.018	0.95%	112
Learning Rate = 0.05, Iterations = 1000	LSM	0.043	2.56%	31
Learning Rate = 0.05, Iterations = 1000	SVM	0.078	3.60%	17

Based on the results presented in Table 6, the following key findings emerge: as the learning rate and number of iterations increase, the nonparametric analysis model based on the BP neural network exhibits significant improvements in both MAE and MRE. This suggests that adjusting these parameters enhances the model’s accuracy. Regardless of the parameter settings, the BP neural network model consistently outperforms the LSM and SVM methods, displaying lower MAE and MRE values, underscoring its superiority in meter coefficient analysis. While the training time of the BP neural network slightly increases with the number of iterations, the associated improvement in accuracy justifies this incremental time cost. These experimental results affirm that the BP neural network model maintains good accuracy and stability under varying parameter settings, emphasizing its effectiveness and robustness in meter coefficient analysis. Furthermore, these findings imply that judicious selection of model parameters can optimize performance to meet diverse practical application requirements.

In conclusion, the nonparametric analysis model based on the backpropagation neural network proposed in this study demonstrates significant superiority in meter coefficient analysis. In comparison to the traditional least squares method, the model achieves an MAE of 0.025 and an MRE of 1.32% on the test dataset, whereas the LSM method records an MAE of 0.043 and an MRE of 2.56%. This denotes a substantial improvement in accuracy with the BP neural network model, highlighting the robust capabilities of nonparametric models in handling complex relationships and nonlinear problems. Additionally, the nonparametric analysis model used in this study, in contrast to traditional methods, does not rely on predefined parameter forms, offering greater flexibility and adaptability. Through deep learning of meter data, the model autonomously identifies and extracts useful features, thereby enhancing the accuracy of meter coefficient analysis. However, this nonparametric analysis model has inherent limitations. Due to its relatively complex structure, it necessitates longer training time and consumes considerable computational resources, limiting its efficiency in practical applications to some extent. Furthermore, the nonparametric nature of the model raises the potential for overfitting, necessitating the implementation of appropriate regularization techniques and cross-validation. Overall, the nonparametric analysis model based on the BP neural network proposed in this study demonstrates commendable performance in meter coefficient analysis, particularly in terms of accuracy. Despite challenges such as extended training time and high computational resource consumption, its excellent performance and potential in addressing complex data issues position it as a valuable reference method in the realm of energy metering and load prediction within the power industry.

## Conclusion

The advancement of information technology has greatly enhanced people’s ability to process information and has become a driving force for the development of various industries. In particular, the non-parametric analysis model of electric meter coefficients has become a vital component in the power industry. Researchers have been focusing on the effective integration of deep learning and neural network technologies and the selection and cleaning of raw data to eliminate noise and outliers. Decision variables such as the level of current transformers, the capacity of phase voltage transformers, and the current transformation ratio are selected in the non-parametric electric meter coefficients analysis model. Data pre-processing, feature extraction, and normalization are then carried out to obtain training and testing datasets. The findings of this study highlight a significant improvement in traditional meter coefficient analysis methods, addressing issues like low accuracy and long processing time through the adoption of a nonparametric analysis model based on backpropagation neural networks. Experimental results reveal that compared to traditional methods like the LSM, the proposed model achieves lower MAE and MRE on the test dataset. Specifically, the MAE value on the test dataset is 0.025, and the MRE value is 1.32%, whereas the corresponding metrics for the LSM method are 0.043 and 2.56%. This validates not only the effectiveness of BP neural networks in nonparametric analysis of meter coefficients but also offers practical insights for the power industry in energy metering and load prediction. While the non-parametric analysis model based on BP neural network proposed in this study demonstrates high accuracy in electricity meter coefficient analysis, it is acknowledged that overfitting is an important factor affecting the model’s generalization ability. By implementing techniques such as cross-validation, regularization, and properly setting stopping criteria during training, the issue of overfitting has been partially mitigated. Future work will continue to explore more efficient techniques and strategies to further enhance the model’s generalization ability, ensuring its stability and reliability in practical applications. However, this study also faces certain limitations, such as extended model training time and high computational resource requirements. To mitigate these issues, future research could focus on the following areas for improvement and expansion: exploring more efficient training algorithms or enhancing existing BP neural network structures to reduce model training time and computational resource consumption, investigating regularization techniques or introducing more sophisticated cross-validation mechanisms to enhance model generalization ability and prevent overfitting, finding the optimal balance between model complexity and learning rate through adjustments in network structures and parameter settings to ensure training stability and analysis accuracy, attempting to apply the model to meter datasets from diverse regions or countries, and exploring potential applications of this model in other similar nonparametric analysis tasks, such as coefficient analysis of water meters or gas meters. In conclusion, while the nonparametric analysis model based on BP neural networks proposed in this study exhibits promising performance in meter coefficient analysis, there remains room for further improvement and expansion. Future research endeavors can not only address existing limitations but also explore the application potential of the model in broader scenarios to advance the development of smart grids and energy management systems.

## Data Availability

The data used to support the findings of this study are included within the article.
